# Socializing the evidence for diabetes control to develop “mindlines”: a qualitative pilot study

**DOI:** 10.1186/s12875-021-01521-w

**Published:** 2021-09-07

**Authors:** John W. Epling, Michelle S. Rockwell, Allison D. Miller, M. Colette Carver

**Affiliations:** 1 Department of Family & Community Medicine, Virginia Tech Carilion School of Medicine and The Carilion Clinic, 1 Riverside Circle, Suite 102, Roanoke, VA 24016 USA; 2 Fralin Life Sciences Institute, Virginia Tech, Blacksburg, VA USA; 3 US Air Force Weapons School, Nellis AFB, NV USA

**Keywords:** Mindlines, Implementation, Evidence-based medicine, Diabetes, Primary care, Practice-based research

## Abstract

**Background:**

Evidence on specific interventions to improve diabetes control in primary care is available, but this evidence is not always well-implemented. The concept of “mindlines” has been proposed to explain how clinicians integrate evidence using specifics of their practices and patients to produce knowledge-in-practice-in-context. The goal of this pilot study was to operationalize this concept by creating a venue for clinician-staff interaction concerning evidence. The research team attempted to hold “mindlines”-producing conversations in primary care practices about evidence to improve diabetes control.

**Methods:**

Each of four primary care practices in a single health system held practice-wide conversations about a simple diabetes intervention model over a provided lunch. The conversations were relatively informal and encouraged participation from all. The research team recorded the conversations and took field notes. The team analyzed the data using a framework adapted from the “mindlines” research and noted additional emergent themes.

**Results:**

While most of the conversation concerned barriers to implementation of the simple diabetes intervention model, there were examples of practices adopting and adapting the evidence to suit their own needs and context. Performance metrics regarding diabetes control for the four practices improved after the intervention.

**Conclusion:**

It appears that the type of conversations that “mindlines” research describes can be generated with facilitation around evidence, but further research is required to better understand the limitations and impact of this intervention.

## Background

Type 2 diabetes mellitus in adults is a leading contributor to morbidity and mortality in the United States and is one of the most prevalent and expensive chronic conditions managed in primary care [1]. Improvement of diabetes control, as measured by the percentage of a patient panel meeting a threshold of diabetes control, is a common measure for quality in primary care and is a Healthy People 2030 goal [2, 3]. This measure has been used on our health system’s “quality scorecard” (used to determine pay-for-performance incentives for system physicians) for the past decade but progress on significantly improving performance on this metric has been elusive.

Prior to the study, the leadership team of the department of Family & Community Medicine in our large health system in the southeast United States had developed an evidence-based intervention framework—the LIVE! Framework—to address the problem of poorly controlled diabetes in the affiliated practices. This framework emphasized four major interventions to reduce average glycosylated hemoglobin (HgbA1c or A1c) in patients with HgbA1c greater than 9%—improved healthy eating and physical activity (L—lifestyle), increased use of insulin to control blood sugars (I—insulin), increased visit frequency (to a goal of every 1–3 months) (V—visit frequency), and attention to comorbid emotional and mood disorders that can worsen diabetes control (E—emotional care). As part of development of the framework, the leadership team had gathered informal feedback from department clinicians and revised the framework accordingly. The feedback revealed clinician concerns about a gulf between the existing evidence-based recommendations and the practice and patient-centered realities – for example, time, resource awareness, and resource availability.

This gulf, or “implementation gap” has been noted and analyzed by many primary care researchers [4, 5]. Gabbay and LeMay have observed, in their qualitative study of primary care practices, that simple awareness of existing evidence and guidelines was not sufficient, but that the evidence needed to be socialized within the practice group, where it was vetted by the members of the practice and seen through the lens of the local context [6]. This “knowledge-in-practice-in-context” is postulated to form the basis of internal, tacit guidelines (called “mindlines”) used by primary care clinicians [7]. This “mindlines” concept is a model derived from careful systematic observation, but has not yet been used as an intervention to enhance evidence adoption. This intervention would consist of making clinicians aware of evidence, providing an opportunity to informally discuss and consider the evidence, and allowing for adaptation the evidence to fit local context and circumstances.

The time available to primary care clinicians to discuss and review new knowledge has decreased due to the pressures of the business of practice, increased electronic health record (EHR) documentation requirements, and issues of work-life balance [8, 9]. This development has the compound consequence of decreasing the ability of a group of clinicians to socialize the needed evidence and guidelines with their colleagues and staff, as well as decreasing the time available to form personal connections with colleagues. This latter effect could be particularly important given the rates of clinician burnout seen today [10].

This pilot project examined the feasibility of an intervention to promote the socialization of the evidence from the LIVE! framework in primary care practices that are struggling with meeting the metric of average HgbA1c < 9.0%.

## Methods

### Setting and sample

The research team consisted of some members of the department leadership team—an experienced family physician (acting as academic detailer), a senior nurse leader (who has a supervisory relationship with many of the nursing staff in the practices), a Doctor of Pharmacy, a care coordinator/diabetes educator, and a research assistant. The research team identified and recruited 4 practices from a department of Family & Community Medicine in a large health system the southeast United States that had the greatest opportunity for improvement on a routinely measured diabetes quality score (percent of patients with diabetes in panel with a HgbA1c < 9%) and were not experiencing a significant staffing shortage or other major stressor at the time.

### Intervention sessions

The research team visited each practice during an all-practice lunchtime meeting at which food was provided. The care coordinator and pharmacist attended either in person or by phone according to their scheduling needs, but the remainder of the team was available in person. Two different research assistants participated in the study, attending two groups each. The LIVE! framework and recommendations were presented to the practice groups along with short handouts with the department resources for implementing the LIVE! recommendations. The groups were encouraged to reflect on challenges, opportunities, and other reactions to the LIVE! framework in their practices. The facilitator/academic detailer explained each LIVE! intervention and reminded the group to focus on challenges and opportunities specific to their practice. These visits were designed as one-time meetings without specific follow up.

### Data collection

The meetings were audio-recorded, and the recordings were transcribed. The research assistants also took field notes during and immediately after the session. The transcripts and field notes were uploaded to an online collaborative mixed-methods software platform (Dedoose) for analysis. Plans for a follow-up survey to the practices after the visit were abandoned given competing demands on the clinicians at the time.

### Ethics and reporting

This study was deemed exempt from human subjects review by the Carilion Clinic Institutional Review Board. Each member of the practice staff was given information about the study in a one-page handout and was informed that participation in the study was entirely optional. The Standards for Reporting Qualitative Research (SRQR) checklist was used to format this report [11].

### Analysis

The research team undertook the qualitative analysis of the transcripts and field notes. The team recorded innovations and strategies used already by the practices to improve their diabetes care as well as the barriers they perceived to effective care. In addition, thematic analysis was used to guide the coding of the data, using themes from Gabbay and LeMay’s “mindlines” work (see Table 1). The team discussed the analysis iteratively over the course of the project, including a discussion of all the results after data collection was completed.

**Table 1:** Primary codes derived from “mindlines” research used to inform the qualitative analysis

**Code Question**
Does the practice accept the evidence as is?
Does the practice adapt the evidence to context?
Does the practice reject evidence due to practice context?
Is there evidence of innovations from practice (evidence or resources)?
Was there discussion between practice members about the evidence?
Was there discussion among clinicians about evidence?
What barriers to implementation of evidence were discussed?
Were there wishes expressed about ways the evidence could be implemented?

To synthesize the results, the research team looked for specific examples of the adoption of elements of the LIVE! Framework and examined barriers to implementing the LIVE! Framework in the practices. The team looked specifically for evidence of the participants in the meeting discussing and adapting the evidence and having the sorts of discussions that would help them develop the appropriate “mindlines” (knowledge-in-practice-in-context) for diabetes care.

In addition, the research team analyzed the results of the diabetes-related quality scorecard measure for each practice for the 3 months before and 3 months after the practice visits. This monthly score notes the number of active patients with diabetes in the practice who have an HgbA1c tested in the past year and for whom that tested result was < 9%. Testing frequency was left up to the discretion of the treating clinician for each patient.

## Results

The four practice visits were completed in June 2019. There was generally near complete attendance of the practice staff at the meetings, though this was not formally assessed. The principal clinicians (physicians, nurse practitioners and physician’s assistants) from each practice were present at each meeting. The practices ranged from large (20 + people, clinicians, and staff) to small (1 or 2 clinicians, < 10 staff) and included both rural and urban/suburban practices.

The practices noted many ways in which they were already engaged with improving their quality scores for the care of patients with diabetes. Some practices saw benefit from some of the technologic features that the health system was implementing. Most practices had been using both clinician-centered and clinic-centered workflow changes prompted in part by their involvement in patient-centered medical home activities in the past. Finally, practices had generally been able to access the limited community resources available to them, sometimes in creative ways. Table 2 reviews some of the details of these practice behaviors.

**Table 2:** Practices reported strategies already in place to improve their quality measures

**Category**	**Strategy Details**
**Technology**	Patient portal usage to adjust insulin
	Utilization of tele-psychiatry (from pilot practice)
**Practice Organization**	Uniform refill and visit policies
	Lab draws (HgbA1c) upon arrival
	Registry use
	Posting and reviewing quality scores
**Clinician innovations**	Huddling
	Chart preparation
	Use of lower-cost insulins (neutral protamine Hagedorn (NPH), etc.)
**Resources**	Medication assistance programs
	Medication discount programs
	Pharmacy technician and Doctor of Pharmacy assistance
	Relationships with outside facilities/consultants
	Applying for insurance coverage for exercise facility membership

The practices noted many barriers to care for their patients. These fell into four main categories: patient factors, local context limitations, cost, and lack of community resources (See Table 3). In general, the discussion of barriers to care dominated the discussions, and in one practice, it was difficult to move the discussion beyond these complaints, limiting both discussion about successful behaviors as well as the mindline-associated behaviors discussed below.

**Table 3:** Practice-reported barriers to care for patients with diabetes

**Barrier**	**Details**
**Patient factors**	Patient does not follow through with referral (have to make their own appointments)
	Patients are reluctant to change medications
	Non-compliant patients who “should not be on our metric list”
	Patient’s agenda is different than the quality agenda
	Patients do not trust people on the phone (issue with telephonic care coordination support)
	Patient desire for one-stop shopping – they are not going to come back to the office multiple times
	Our patients are sicker, more disadvantaged, etc
	Lack of transportation
**Context Factors**	Patient load
	Care coordinators are not on site any longer
	Lack of reliable information transfer between institutions
	Practice staff too lean (especially nursing)
	Lack of access to medication samples
	Difficulty being an outlying clinic (“away from mothership,” “outcast”)
	Short visit times/crowded agendas
	Lack of RN (Registered Nurse) time for insulin teaching
	Quality agenda overrides other patient-centered care
**Resources**	Inability to refer poorly controlled patients to endocrinology
	Lack of nutrition referrals
	Community resources insufficient
**Cost**	Medication costs, lack of coverage for non-insulin options
	Clinicians do not always know costs

To analyze whether these practice visits could generate the type of interdisciplinary practice interactions necessary for “mindlines” generation, the research team looked for 1) discussion of the evidence (the LIVE! recommendations) between practice members, 2) evidence that the practice either accepted the new evidence or engaged in adapting the recommendations to their own context, 3) if they rejected any parts of the evidence due to their practice context, and 4) if they expressed any wishes about a change in external circumstances that would allow them to achieve better quality in treating their patients with diabetes. We found examples of each of these phenomena, but the discussion of barriers to implementation of each of the aspects of the LIVE! framework dominated the discussion. The relevant excerpts of these conversations are presented in Table 4.

**Table 4:** Evidence of “mindlines” conversations during practice visits

**Type of “mindlines” conversation**	**Excerpts**
**Discussion among practice members**	Interaction with each other about the registry and how they can work with each other to improve. [Field Note]
	No formal meetings: “We constantly have conversations. Fine tuning, tweaking, ever-changing model” PA [Physicians Assistant] and Doc meet weekly and discuss Take 3 [a local literature review newsletter] – friendly educational session. Doc and PA seem on same page, seems mutually respectful. Complete each other’s sentences. They acknowledge each other’s different styles but prioritize care pattern consistency. [Field Note]
	Female5: “I would like to see who is on the registry I am associated with.” Female6: “I have a big ole stack of them. [Laughing]” Female5: “Good I would like to see it.” Female 3: “I have a registry on my desk, just printed a brand new one.” [Transcript]
	When female doc said local counselors are really good, the rarely-talking male doc shook head in disagreement rigorously; Disagrees; Silent docs looking down even when solicited for comments (about mental health) [Field Note]
	Female4: “I have a list of counselling services available in the area, and I have a friend who is a psychiatrist in another area that has given me resources… If you ever need any, I have a list.” Facilitator: “So, how does that information get shared across the practice?” Female4: “I printed it out, cut it out and gave it to front desk for people or if we need a counselor. It was put in the referral area. It was available for anybody that needs it.” [Transcript]
**Practice accepts new evidence**	Male 5: “Probably [see them] every 3 months or so, or 3–6 months if it’s over 9 you said?” Facilitator: “Over 9.” Male 5: “Over 9 probably every 3 months.” [Transcript]
	“Yes, I have changed their depression medicine as well as their diabetes medication, plus improved their diabetes.” [Transcript]
	“I have a lot of success stories; I have a lot of patients I have dealt with an A1C greater than 9 and metformin and other medications and switching medications and adding agents and it has cut A1C in half.” [Transcript]
	“We look at quality metrics and we have started to print list and that type of thing.” [Transcript]
	“One of our care coordinators worked in psychiatry and actually did a lot of counseling and so we do that. We also find resources in the community but there is another opportunity with tele-medicine and behavioral health as well.” [Transcript]
	Screen for depression, most diabetics already on anti-depressant, it is addressed in follow-up [Field Note]
	Try to get people in for diabetic self-management or diabetic education [Field Note]
**Practice adapts new evidence**	“That’s one of the secrets that I’ve learned coming here is using the NPH instead of the long-acting ones.” [Transcript]
	Constantly fine tuning and tweaking and trying things Interaction with each other about the registry and how they can work with each other to improve. [Field Note]
	“Sometimes switch them to NPH or 70/30 something that is less expensive.” [Transcript]
	“With counseling we don’t make those appointments any longer, because of the fact we were making them in the beginning and the back and forth of where they’re calling the patient and the patient was like I don’t need this, so they were like we are calling all these patients and they don’t want any of this help so now we give them the information and you contact them and then they don’t contact them.” [Transcript]
	Female 16: “Because I am the one, they see at the beginning and I am the one they see for the lab and I am the one they see at check out. So, I am pretty much everything or we are pretty much everything. So, we are the lab and the ones that are checking them in and telling them what the doctor wanted to do…once again they dump it all in the lab. They tell you a lot of things” Facilitator: “They do? I feel like there is potential here. What if we surrounded her with some sort of resources to be able…” Female 16: “What’s another job title I could have?” [Transcript]
**Practice rejects new evidence**	“I honestly, just because of the complexity of it [referral to care coordinator], I almost never do that. I think I have done it one time in 10 months.” [Transcript]
	When PI [Principal Investigator, Facilitator] shared resource page, two staff adamantly pointing at page and saying no. [Field Note]
	“But I know it’s tele, I think it’s horrible actually, but I mean that’s what’s done so tele-everything. I don’t like it. I think that, that’s what I’m trying to say from the beginning, being in the room with a patient, just trying to understand where they are at. I don’t’ think you can get that over a video screening. I don’t like that at all. That’s what I say about that.” [Transcript]
**Wishes**	“I have talked to management about having a nutrition class on Saturday once a month and I would want to do that but how do you make a nutrition class that everyone in our area can attend and would comprehend it?” [Transcript]
	“What would be nice is if we could have, when they came in for their appointment with us for a comprehensive appointment if we have a care coordinator, “Oh hey, I think, you know what? Why don’t you meet with her while you are here? It’s a one stop shop, we can talk about our diet and your medications more thoroughly than the 15-min visit you had with your provider.” [Transcript]
	“This is like I don’t know, but it would be nice to have counseling available in area. A person that I could send people to. You can wish for all kinds of things.” [Transcript]

**Fig. 1:**
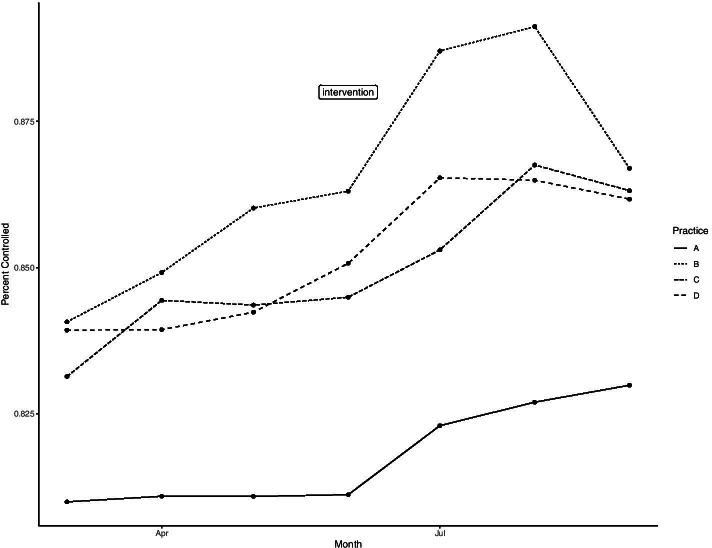
HgbA1c quality measure performance by practice before and after intervention

The practices’ performance on the diabetes control measure (percent of practice with diabetes whose HgbA1c is less than 9%) improved slightly over the course of the study (Fig. 1) but given the relatively few data points, formal trend analysis was not undertaken.

## Discussion

The results of this study show that a one-time, informal, practice discussion around evidence-based interventions can generate the kinds of discussions that may lead to the development of “mindlines.” Clinicians, nurses, and clerical staff engaged each other about novel ways to implement the evidence informed each other about existing practices and shared both barriers and potential solutions to implementation. Because of some recent changes in how nurse care coordination was delivered to the patients in the health system’s accountable care organization infrastructure, the discussions were dominated by some complaints and barriers referable to that change, however, each of the groups did show some evidence of “mindlines-generating” discussions of the evidence. Facilitating these sorts of conversations about the evidence routinely in practice has the potential to develop and strengthen clinicians’ internal “mindlines” around evidence-based practice.

The change in the performance on the diabetes measure was encouraging, but far from conclusive as there were too few data points to allow for meaningful trend analysis. As this was a secondary outcome, we did not compare this change to the other practices in the department, but this measure had been difficult to change in practices over the previous several years. Secular trends and/or an approaching metric deadline (the end of the fiscal year on September 30^th^) may have accounted for the rise in scores.

More work is needed to operationalize the “mindlines” framework laid out by Gabbay and LeMay, and because this is a primarily qualitative study, its findings must be evaluated in a more rigorous manner before implementation at scale. Previous work in this area by Crabtree, et al., finds that “People working in practices are well educated and want to do well; however, they need support in finding ways to interact and collaborate with colleagues…most practices are resistant to protecting time for reflection…thus, “forcing” time and space for reflection may be one of the more important components of a change management strategy” [12]. A conceptual framework of barriers to evidence implementation derived from a systematic review of reviews includes many of the concepts of socialization, context and adaptability that are essential to the “mindlines” model and provide a solid theoretical foundation for deriving potential interventions such as this one [13]. In this era of increasing healthcare clinician burnout, interventions that involve interdisciplinary, relaxed, problem solving communication can re-establish and improve the social connectedness of outpatient practices, which may reduce burnout and improve practice performance [10, 14].

## Conclusion

An intervention aimed at generating unstructured interdisciplinary conversations about evidence-based interventions to improve diabetes control is feasible in primary care practices. The resulting interdisciplinary conversations included discussions of adaptation and adoption that could influence the development of “mindlines” among the practice clinicians and staff. Future research plans in this area include a regular schedule of these meetings with incentives to maintain practice-wide participation and fine-tuning the presentation of the evidence.

## Data Availability

Data is not available due to the presence of general details and information that could render the practices identifiable.

## Abbreviations

LIVE!: An acronym used for the diabetes care framework – lifestyle, insulin, visit frequency and emotional care
HgbA1c or A1c: Hemoglobin A1c or glycosylated hemoglobin
SRQR: Standards for Reporting Qualitative Research
NPH: Neutral protamine Hagedorn insulin
RN: Registered Nurse
PA: Physician’s assistant
PI: Principal Investigator, facilitator of discussion groups
